# *Cunninghamella* as a Microbiological Model for Metabolism of Histamine H_3_ Receptor Antagonist 1-[3-(4-*tert*-Butylphenoxy)propyl]piperidine

**DOI:** 10.1007/s12010-012-9880-8

**Published:** 2012-09-16

**Authors:** Elżbieta Pękala, Paulina Kubowicz, Dorota Łażewska

**Affiliations:** Department of Technology and Biotechnology of Drugs, Faculty of Pharmacy, Jagiellonian University Medical College, Cracow, Poland

**Keywords:** Cunninghamella, Biotransformation, Microbiological model, In vitro metabolism, In silico metabolism

## Abstract

The aim of the study was to analyze the ability of the microorganism *Cunninghamella* to carry out the biotransformation of 1-[3-(4-*tert*-butylphenoxy)propyl]piperidine (DL76) and to compare the obtained results with in silico models. Biotransformation was carried out by three strains of filamentous fungus: *Cunninghamella echinulata*, *Cunninghamella blakesleeana*, and *Cunninghamella elegans*. Most probable direction of DL76 metabolic transition was the oxidation of the methyl group in the *tert*-butyl moiety leading to the formation of the metabolite with I° alcohol properties. This kind of reaction was conducted by all three strains tested. However, only in the case of *C. blakesleeana* that biotransformation product had a structure of carboxylic acid. CYP2C19 was identified by Metasite software to be the isoform of major importance in the oxidation process in the *tert-*butyl moiety of DL76. In silico data coincide with the results of experiments conducted in vitro. It was confirmed that *Cunninghamella* fungi are a very good model to study the metabolism of xenobiotics. The computational methods and microbial models of metabolism can be used as useful tools in early ADME-Tox assays in the process of developing new drug candidates.

## Introduction

Each drug before it is introduced to the market must undergo a complicated procedure. At the beginning, it must be checked if a given structure is active; if yes, it is not the end of the procedure. The metabolism of such a substance and its toxicity is also very important [1]. Before the drug reaches to clinical trials and is administered to humans, there is another stage—preclinical screening [2]. This stage gives us information about ADME(T)—absorption, distribution, metabolism, excretion, and toxicology [3].

Drug metabolism studies can rely on the use of animal systems like mouse [4], rat [5], or guinea pig [6] (in vivo). Unfortunately, these models suffer from a number of limitations like ethical aspects, time that must be spend on breeding animals, and last, but not least, cost of experimental models. Because of these disadvantages, use of in vitro studies becomes an important tool for testing drugs and for production of valuable drug metabolites. In vitro screening assays include human liver models, like perfused liver [7], cell lines [8], hepatocytes [9], and liver slices [10]. S9 fractions are very interesting because they contain both phase I and phase II activities and are useful in the study of xenobiotic metabolism and drug interaction [11]. Human liver microsomes are another popular in vitro model [12]. They are a rich source of drug-metabolizing enzymes [13]. However, in vitro studies are not only on human liver models. To elucidate the metabolism of xenobiotics, microbial cultures like bacteria [14], fungi [15], or yeast [16] can be used. The cost of in vitro models is lower than in vivo [17], culture and extraction of cytochrome P450 are simple [18], and in such models, the so called “non-suffering organisms” are used.

Another way to study the metabolism of a drug is the so called in silico model. It allows to predict the biological properties and parameters of ADME(T) by computational simulation [19]. However, neither in vitro models—cell or tissue culture, microsomal preparations—nor in silico studies will replace animal system, but the number of animals suffering from such tests may, or even must, be limited. One of such widely used in vitro microbiological model is *Cunninghamella* [20].

*Cunninghamella* has specific properties which make this fungus very useful in studies of drug metabolism. This property is the ability to metabolize a wide variety of drugs, over a hundred of them, in manners that are similar to those in mammalian enzyme systems [20]. It was proven that *Cunninghamella* has enzymes that are synonymous to those involved in xenobiotic detoxification in mammals [21]. Moreover, there are many evidences [21, 22] that *Cunninghamella* can predict the fate of the drug in the mammalian organism better than other microorganisms.

The histamine H_3_ receptor (H_3_R) has been identified in the central nervous system (CNS) and peripheral nervous system as a pre-synaptic receptor controlling the release of histamine and numerous other neurotransmitters [23]. In the past, histamine H_3_R antagonists were imidazole-containing compounds. Imperfection of these structures appeared in unwanted hepatic cytochrome P450 inhibition and potential drug–drug interactions. Therefore, a new class of non-imidazole histamine H_3_R antagonists was designed and synthesized [24]. Potential therapeutic use of histamine receptor ligands involves treatment of CNS diseases [25]. In our department for many years, we were looking for new ligands of the H_3_R in a group of non-imidazole derivatives. One of the newly synthesized compounds, 1-[3-(4-*tert*-butylphenoxy)propyl]piperidine—DL76, was proved to be highly potent and orally available histamine H_3_ receptor antagonist (*h*H_3_R *K*_i_ = 22 ± 3 nM—affinity for the recombinant human H_3_R, stably expressed in CHO; ED_50_: 2.8 ± 0.4 mg/kg) [26].

**Fig. 1:**
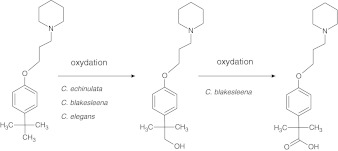
Possible direction of DL76 metabolism carried out by *Cunninghamella*

Referring to several studies confirming that microbial *Cunninghamella* model is able to carry out metabolism in a manner similar to humans [20], we decided to check DL76 biotransformation pathway in in vitro model using different strains of *Cunninghamella* (Fig. 1). The report also compares the results obtained from the microbial biotransformation of the DL76 to the in silico model performed by a metabolism-simulating program Metasite.

## Materials and Methods

### Substrates and Chemicals

The media required for the growth of *Cunninghamella* strains were purchased from BioShop (Canada) (potato dextrose agar) and from Sigma-Aldrich (St Louis, MO, USA) (CSL, Corn Steep Liquor). DL76 was synthesized in the Department of Chemical Technology and Biotechnology of Drugs, Faculty of Pharmacy, Medical College of Jagiellonian University, Cracow, Poland. HPLC-grade acetonitrile, dichloromethane, water, and formic acid were obtained from Merck (Darmstadt, Germany). All other chemicals were of analytical reagent grade and were obtained from Sigma-Aldrich.

### Microorganisms and Culture Conditions

*Cunninghamella echinulata* NRRL 1384 (which was a gift from A.J. Carnell, University of Liverpool, UK), *Cunninghamella blakesleeana* DSM 1906, and *Cunninghamella elegans* DSM 1908 (Deutsche Sammlung von Mikroorganismen und Zellkulturen GmbH, Braunschweig, Deutschland) were propagated on potato dextrose agar (PDA) plate at 30 °C for 7 days. A fermentation basal medium (20-g d-glucose and 20-g corn step liquor in 1,000 ml of water at pH 5.0) was seeded with a 200-μl suspension of spore gained by wetting colonies on solid medium PDA with sterile water.

### Biotransformation Experiments

Biotransformation was carried out in a 50-ml fermentation medium, which was inoculated with 200-μl suspension of corresponding *Cunninghamella* species. Microorganisms were cultured in 30 °C for 48 h. Flasks were shaken for the whole time. After 2 days, 250 μl of the stock solution, which was prepared by dissolving 100 mg of DL76 in 1 ml DMSO (the use of co-solvent was dictated by the poor solubility of the substance in water; Log *P* = 5.09), was added to the fermentation medium. The biotransformation was carried out for 7 days, and its progress was followed using thin layer chromatography (TLC) and liquid chromatography electrospray ionization-tandem mass spectrometry (LC-MS/MS).

### Extraction of Products

During the subsequent days of biotransformation, 500-μl samples were taken from all cultures. Each sample was extracted with methylene chloride. The water phase was separated from organic phase. In the following experiment, only organic phase was used, which next was dried over anhydrous sodium sulfate and concentrated in vacuo.

The cultures, after a 7-day biotransformation of DL76, were also extracted with methylene chloride, and the whole organic phase after concentrated in vacuo was used to the following studies.

### Analytical Methods

For the identification of compound DL76 and its possible biotransformation products, both TLC and LC-MS/MS were applied. TLC was performed using Merck aluminum plates coated with silica gel with a thickness of 0.2 mm. It was concluded, as a result of many experimental studies, that the most preferred developing system is a mixture of methylene chloride and methanol (9:1 + 5 drops of NH_3_). Observation of the chromatograms was carried out under UV light. The chromatograms were also stained with iodine.

### Liquid Chromatographic Conditions

A liquid chromatography for determination of DL76 and its possible metabolites was performed using an Agilent 1100 HPLC (Agilent Technologies, Waldbronn, Germany) system consisting of a degasser, binary pump, a column oven, and an autosampler. Chromatographic separation was carried on an Xbridge^TM^ C18 analytical column (2.1 × 30 mm, 3.5 μm;, Waters, Dublin, Ireland) with the oven temperature set at 30 °C. The mobile phase of acetonitrile and water with an addition of 0.1 % formic acid with gradient elution was set at flow rate of 0.3 ml. A sample volume of 10 μl was injected into LC-MS/MS system.

As a detector, triple quadrupole mass analyzer API 2000 from Applied Biosystems Sciex MDX (Concorde, Ontario, Canada) was used. Ionization was performed using the EPI (Elektrospray Ionization).

### Mass Spectrometric Conditions

Mass spectrometric detection was performed on an Applied Biosystems MDS Sciex (Concord, Ontario, Canada) API 2000 triple quadrupole mass analyzer equipped with an electrospray ionization (ESI) interface. ESI ionization was performed in the positive ion mode. The ion source temperature was maintained at 450 °C. The ion spray voltage was set at 5,500 V. The curtain gas (CUR) was set at 10 V and the collision gas (CAD) at 12 V. The optimal collision energy was 50 V. Data acquisition and processing were accomplished using the Applied Biosystems Analyst version 1.4.2 software.

### Proton Nuclear Magnetic Resonance and IR spectra

Proton nuclear magnetic resonance (^1^ H NMR) spectra were obtained in a Varian Mercury spectrometer (Varian Inc., Palo Alto, CA, USA), in CDCl_3_, operating at 300 MHz. Chemical shifts are reported in δ values (in parts per million) relative to TMS δ = 0 (^1^ H), as internal standard. The *J* values are expressed in Hertz (Hz). Signal multiplicities are represented by the following abbreviations: s (singlet), brs (broad singlet), d (doublet), t (triplet), and m (multiplet). The IR spectra were recorded on a Jasco FT/IR 410 spectrometer (KBr pellets).

### In Silico Studies

Computational simulation of metabolism of the title compound was carried out by use the MetaSite software, demo version 2.1.0. (2005), Molecular Discovery Ltd.

MetaSite is a computational procedure to predict metabolism issues related to cytochrome-mediated reactions in phase I metabolism. The methodology uses 3-D maps of interaction energies between the protein, chemical probes, and the 3-D structure of the compounds to be analyzed. MetaSite procedure is completely automated and does not require any user assistance. All the work can be handled and submitted in batch queue. The basic concept of MetaSite is to compare the interaction patterns inside the protein with the 3-D structure of the ligand. The information obtained from a friendly user interface can be easily translated into decisions in the drug discovery process.

## Results and Discussion 

### In Vitro Biotransformation Study

In our study, three strains of filamentous fungus were used: *C. echinulata* NRRL 1348, *C. blakesleeana* DSM 1906, and *C. elegans* DSM 1908.

**Fig. 2:**
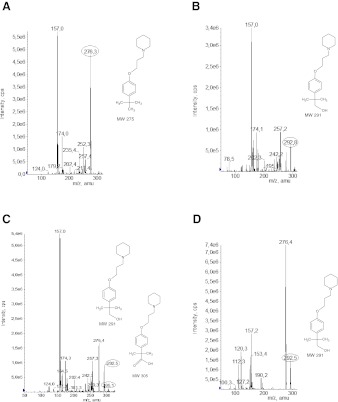
**a** LC-MS/MS spectrum of DL76. The LC-MS/MS spectrum of metabolites of DL76 obtained after biotransformation carried out by **b** *C. echinulata* NRRL 1384, **c** *C. blakesleeana* DSM 1906, and **d** *C. elegans* DSM 1908

During the study, the evidence that all strains are able to carry out the biotransformation of DL76, novel histamine H_3_ receptor antagonist, were obtained. The samples from all cultures were taken in the subsequent days of biotransformation, and TLC was performed. The following *R*_f_ (retardation factor) values were obtained: 0.50–0.52 for DL76 and 0.34–0.36 for its metabolites. It was noticed that in seventh day of biotransformation, no new derivatives appeared. After completion of the reaction, samples were taken to investigate the structure of the obtained derivatives of DL76. LC-MS/MS spectra were done. In the mass spectrum of DL76, protonated molecule [M + H]^+^ at *m/z* 276 (Fig. 2a) was detected, which corresponds to the molecular weight (275) of the parent molecule. In the mass spectra of DL76 biotransformation products, protonated parent molecule ([M + H]^+^ at *m/z* 276), its alcoholic and acidic metabolites (protonated ion [M + H]^+^ at *m/z* 292 and *m/z* 306, respectively) were noticed (Fig. 2b–d).

We can assume, according to the LC-MS/MS spectra that the most probable direction of metabolic transition of DL76 was the oxidation of the methyl group in the *tert-*butyl moiety leading to the formation of a metabolite with alcohol I° properties (Fig. 2b–d). This kind of reaction was biocatalised by all three strains (Fig. 1). However, only in the case of strain *C. blakesleeana* that biotransformation product had the molecular ion corresponding to carboxylic acid (Figs. 1 and 2c).

*C. echinulata* NRRL 1384 was able to carry out the biotransformation in 100 %; there was no trace of the presence of protonated ion at *m/z* 276 for DL76 (Fig. 2b). The level of biotransformation carried out by *C. elegans* DSM 1908 was the lowest (Fig. 2d).

^1^ H NMR and IR spectra for D76 and its metabolite after biotransformation using *C. echinulata* NRRL 1384 were done. The ^1^ H NMR spectra of DL76 represented one distinct singlet which appeared at δ 1.34 for nine protons confirming the presence of three methyl groups, the *tert*-butyl substituent. While in the ^1^ H NMR spectra of DL76, metabolite singlet at δ 1.39 was detected, suggesting only six protons for two methyl groups. Additionally, other singlet signals at δ 2.0 for one proton from hydroxyl group and at δ 3.78 for two protons of methylene group (−CH_2_OH) were noticed. These last two singlets in the ^1^ H NMR spectra confirmed that biotransformation of D76 leads to oxidation of one of the three methyl groups in the *tert*-butyl substituent. Also, the IR spectra of DL76 metabolite confirmed the presence of hydroxyl functional group at 3,420 (OH). This group was not present in the IR spectra of DL76. Biotransformed product of DL76 was determined as I^o^ alcohol: 2-methyl-2-(4-(3-(piperidin-1-yl)propoxy)phenyl)propan-1-ol.

DL76: 1-(3-(*tert*-butylphenoxy)propyl)piperidine

^1^ H NMR (300 MHz, CDCl_3_): δ 1.34 (s, 9 H, 3 x CH_3_), 1.50 (s, 6 H, 3 x CH_2_ of piperidine), 1.81 (m, 2 H, CH_2_ ), 2.24 (t, 4 H, 2 x N-CH_2_ of piperidine, *J* = 6.54 Hz), 2.36 (m, 2 H, N-CH_2_), 3.39 (t, 2 H, O-C H_2_, *J* = 6.32 Hz), 6.69 (d, 2 H, ArH, *J* = 7.50 Hz), 7.18 (d, 2 H, ArH, *J* = 7.74 Hz). IR (per centimeter): 2,953, 2,931, 2,867, 2,770, 1,610, 1,513, 1,466, and 1,230.

Metabolite of DL76: 2-methyl-2-(4-(3-(piperidin-1-yl)propoxy)phenyl)propan-1-ol)

^1^ H NMR (300 MHz, CDCl_3_): δ 1 .39 (s, 6 H, 2 x CH_3_), 1.50 (s, 6 H, 3 x CH_2_ of piperidine) 1.81 (m, 2 H, CH_2_ ), 2.0 (bs, 1 H, OH ), 2.23 (t, 4 H, 2 x N-CH_2_ of piperidine, *J* = 6.74 Hz), 2.36 (m, 2 H, N-CH_2_), 3.78 (s, 2 H, CH_2_OH), 3.94 (t, 2 H, O-CH_2_, *J* = 6.20 Hz), 6.65 (d, 2 H, ArH, *J* = 7.45 Hz), 7.20 (d, 2 H, ArH, *J* = 7.70 Hz). IR (per centimeter): 3,420, 3,005, 2,917, 1,646, 1,435, 1,406, and 1,224.

### In Silico Biotransformation Study

Nowadays, different computational approaches are used to predict the position of metabolism [27–30]. These approaches can be grouped into quantitative structure–activity relationship-based, pharmacophore-based, structure-based (docking), reactivity-based, and rule-based methods.

**Fig. 3:**
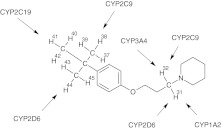
Metabolic simulation of DL76 performed by MetaSite for isoforms of CYP450

In our in silico study, MetaSite software was used to predict DL76 metabolic transformation and particularly to help in the identification of the metabolite structure. MetaSite is able to predict human CYP1A2, CYP2C9, CYP2C19, CYP2D6, and CYP3A4 regioselective metabolism using only the 3-D structure of the given compound. The recognition of the site of metabolism could be a significant step in designing new compounds with a better pharmacokinetic profile. Labile compounds can be stabilized when the site of metabolism is known by adding stable groups at metabolically susceptible positions. MetaSite program does not give the structures of metabolites that are formed but, by analyzing three-dimensional structure of molecules, only suggests a probable site of attack of the enzyme. The site of metabolism is described by a probability function (*P*_SM_) which is correlated to, and can be considered to be, an approximation of the free energy of overall process [27]. Figure 3 and Table 1 show the MetaSite ranking of the probability of metabolism for the different molecular positions. The MetaSite software applied to DL76 predicted sites of oxidation by CYP2C19 in *tert*-butyl moiety: H-37, H-38, H-39, H-40, H-41, H-42, H-43, H-44, and H-45 (*P*_SM_ = 1.43) (Fig. 3). This result coincides with the data of conducted experiment using *Cunninghamella* as a model to study the metabolism of xenobiotics. MetaSite program moreover indicated the methylene group in alkyl chain which is adjacent to the nitrogen atom of piperidine (H-31, H-32) as the very probable site of oxidation process carried out by CYP1A2, CYP3A4, and CYP2D6 (Fig. 3). The predicted value of *P*_SM_ for CYP3A4 (1.7) and CYP1A2 (1.65) indicated that those two isoforms might have the greatest importance in metabolic pathway of given compound (Table 1), while CYP2C9 is the isoform which has the least importance in the metabolism of methylene group (H-31, H-32) of DL76.

**Table 1:** Value of the probability function (*P*_SM_) for selected DL76 atoms

CYP3A4		CYP1A2		CYP2D6		CYP2C9		CYP2C19	
Atom	*P*_SM_	Atom	*P*_SM_	Atom	*P*_SM_	Atom	*P*_SM_	Atom	*P*_SM_
H-37		H-37		H-37		H-37		H-37	
H-38	0.5916	H-38	0.2694	H-38	0.6066	H-38	0.9166	H-38	1.4333
H-39		H-39		H-39		H-39		H-39	
H-40		H-40		H-40		H-40		H-40	
H-41	0.5916	H-41	0.2694	H-41	0.6066	H-41	0.9166	H-41	1.4333
H-42		H-42		H-42		H-42		H-42	
H-43		H-4∙3		H-43		H-43		H-43	
H-44	0.5916	H-44	0.2694	H-44	0.6066	H-44	0.9166	H-44	1.4333
H-45		H-45		H-45		H-45		H-45	
H-31	1.7000	H-31	1.6500	H-31	1.6000	H-31	1.2816	H-31	1.2225
H-32		H-32		H-32		H-32		H-32	

The oxidative and reductive capabilities of microorganisms, especially fungi, have been well known for a long time and are used in preparative reactions. Among the fungi, *Cunninghamella* species have the ability to metabolize a wide variety of xenobiotics in manners that are similar to those in mammalian enzyme systems [31–33]. The genus *Cunninghamella* contains species of importance in medical mycology and in biotechnological processes. *Cunninghamella bertholletiae*, *C. elegans*, and *C. echinulata* are the most common species.

Results of our research show similar direction of biotransformation reactions for DL76 and terfenadine, described by Mazier et al. [34]. The *tert*-butyl moiety is the same for both molecules, and the methyl group in the *tert*-butyl substituent is in both compounds very sensitive to oxidation process. For the synthesis of active metabolite fexofenadine, scientists used *C. blakesleeana* and another type of microbial organisms. The transformation of terfenadine was carried out via oxidation of one of the methyl groups in the *tert*-butyl substituent into alcohol and finally into carboxylic acid. The process of obtaining fexofenadine from terfenadine by using microbial models was patented [34]. The results of our studies confirm that *Cunninghamella* is a suitable model to study the metabolism of compounds that have a chance to become a drug.

In conclusion, identification of the probable metabolites and the sites where molecular metabolism could occur, using LC-MS/MS and MetaSite, can provide chemists with knowledge that would help them in optimizing the structure of the compound in order to improve the metabolic stability of new drug candidates.

## References

[1] de Oliveira CC, Abud AP, de Oliveira SM, Guimaraes FD, de Andrade LF, di Bernardi RP, Coletto EL, Kuczera D, da Lozzo EJ, Goncalves JP, Trindade ED, Buchi DD (2011). Developments on drug discovery and on new therapeutics: highly diluted tinctures act as biological response modifiers. BMC Complementary and Alternative Medicine.

[2] Asha S, Vidyavathi M (2010). Role of human liver microsomes in in vitro metabolism of drugs-a review. Applied Biochemistry and Biotechnology.

[3] Smith DA (2011). Discovery and ADMET: Where are we now. Current Topics in Medicinal Chemistry.

[4] van Waterschoot RA, Schinkel AH (2011). A critical analysis of the interplay between cytochrome P450 3A and P-glycoprotein: recent insights from knockout and transgenic mice. Pharmacological Reviews.

[5] Srinivas NR (2010). Altered disposition of drugs in acute renal failure rat models: drug development strategies and perspectives. Arzneimittel-Forschung.

[6] Gupta UD, Katoch VM (2009). Animal models of tuberculosis for vaccine development. Indian Journal of Medical Research.

[7] Brandon EF, Raap CD, Meijerman I, Beijnen JH, Schellens JH (2003). An update on in vitro test methods in human hepatic drug biotransformation research: pros and cons. Toxicology and Applied Pharmacology.

[8] Donato MT, Lahoz A, Castell JV, Gomez-Lechon MJ (2008). Cell lines: a tool for in vitro drug metabolism studies. Current Drug Metabolism.

[9] Sahi J, Grepper S, Smith C (2010). Hepatocytes as a tool in drug metabolism, transport and safety evaluations in drug discovery. Current Drug Discovery Technologies.

[10] Komatsu T, Yamazaki H, Shimada N, Nakajima M, Yokoi T (2000). Roles of cytochromes P450 1A2, 2A6, and 2C8 in 5-fluorouracil formation from tegafur, an anticancer prodrug, in human liver microsomes. Drug Metabolism and Disposition.

[11] Sumida K, Ooe N, Nagahori H, Saito K, Isobe N, Kaneko H, Nakatsuka I (2001). An in vitro reporter gene assay method incorporating metabolic activation with human and rat S9 or liver microsomes. Biochemical and Biophysical Research Communications.

[12] Eichelbaum M, Burk O (2001). CYP3A genetics in drug metabolism. Nature Medicine.

[13] Lake BG, Price RJ, Giddings AM, Walters DG (2009). In vitro assays for induction of drug metabolism. Methods in Molecular Biology.

[14] Fujita K, Kamataki T (2002). Genetically engineered bacterial cells co-expressing human cytochrome P450 with NADPH-cytochrome P450 reductase: prediction of metabolism and toxicity of drugs in humans. Drug Metabolism and Pharmacokinetics.

[15] Cheng J, Wan DF, Gu JR, Gong Y, Yang SL, Hao DC, Yang L (2006). Establishment of a yeast system that stably expresses human cytochrome P450 reductase: application for the study of drug metabolism of cytochrome P450s in vitro. Protein Expression and Purification.

[16] Srisailam K, Veeresham C (2010). Biotransformation of celecoxib using microbial cultures. Applied Biochemistry and Biotechnology.

[17] Pritchard MP, McLaughlin L, Friedberg T (2006). Establishment of functional human cytochrome P450 monooxygenase systems in Escherichia coli. Methods in Molecular Biology.

[18] Guengerich FP, Martin MV (2006). Purification of cytochromes P450: products of bacterial recombinant expression systems. Methods in Molecular Biology.

[19] Khan MT (2010). Predictions of the ADMET properties of candidate drug molecules utilizing different QSAR/QSPR modelling approaches. Current Drug Metabolism.

[20] Asha S, Vidyavathi M (2009). Cunninghamella–a microbial model for drug metabolism studies–a review. Biotechnology Advances.

[21] Prior JE, Shokati T, Christians U, Gill RT (2010). Identification and characterization of a bacterial cytochrome P450 for the metabolism of diclofenac. Applied Microbiology and Biotechnology.

[22] Sun L, Huang HH, Liu L, Zhong DF (2004). Transformation of verapamil by Cunninghamella blakesleeana. Applied and Environmental Microbiology.

[23] Gemkow MJ, Davenport AJ, Harich S, Ellenbroek BA, Cesura A, Hallett D (2009). The histamine H3 receptor as a therapeutic drug target for CNS disorders. Drug Discovery Today.

[24] Szafarz M, Szymura-Oleksiak J, Lazewska D, Kiec-Kononowicz K (2011). LC-MS-MS Method for the Analysis of New Non-Imidazole Histamine H(3) Receptor Antagonist 1-[3-(4-tert-Butylphenoxy)propyl]piperidine in Rat Serum-Application to Pharmacokinetic Studies. Chromatographia.

[25] Kim SK, Fristrup P, Abrol R, Goddard WA (2011). Structure-based prediction of subtype-selectivity of Histamine H3 receptor selective antagonists in the clinical trials. Journal of Chemical Information and Modeling.

[26] Lazewska D, Ligneau X, Schwartz JC, Schunack W, Stark H, Kiec-Kononowicz K (2006). Ether derivatives of 3-piperidinopropan-1-ol as non-imidazole histamine H3 receptor antagonists. Bioorganic & Medicinal Chemistry.

[27] Cruciani G, Carosati E, De BB, Ethirajulu K, Mackie C, Howe T, Vianello R (2005). MetaSite: understanding metabolism in human cytochromes from the perspective of the chemist. Journal of Medicinal Chemistry.

[28] Singh SB, Shen LQ, Walker MJ, Sheridan RP (2003). A model for predicting likely sites of CYP3A4-mediated metabolism on drug-like molecules. Journal of Medicinal Chemistry.

[29] Ridderstrom M, Zamora I, Fjellstrom O, Andersson TB (2001). Analysis of selective regions in the active sites of human cytochromes P450, 2C8, 2C9, 2C18, and 2C19 homology models using GRID/CPCA. Journal of Medicinal Chemistry.

[30] Lewis DF, Dickins M, Eddershaw PJ, Tarbit MH, Goldfarb PS (1999). Cytochrome P450 substrate specificities, substrate structural templates and enzyme active site geometries. Drug Metabolism and Drug Interactions.

[31] Keum YS, Lee YH, Kim JH (2009). Metabolism of methoxychlor by Cunninghamella elegans ATCC36112. Journal of Agricultural and Food Chemistry.

[32] Amadio J, Murphy CD (2011). Production of human metabolites of the anti-cancer drug flutamide via biotransformation in Cunninghamella species. Biotechnology Letters.

[33] Zhang D, Freeman JP, Sutherland JB, Walker AE, Yang Y, Cerniglia CE (1996). Biotransformation of chlorpromazine and methdilazine by Cunninghamella elegans. Applied and Environmental Microbiology.

[34] Mazier C, Jaouen M, Sari MA, Buisson D (2004). Microbial oxidation of terfenadine and ebastine into fexofenadine and carebastine. Bioorganic & Medicinal Chemistry Letters.

